# Supplementary material for: Collaborative intelligence in AI: Evaluating the performance of a council of AIs on the USMLE
Source: PLOS Digit Health. 2025 Oct 9;4(10):e0000787. doi: 10.1371/journal.pdig.0000787 (PMC12510544; doi:10.1371/journal.pdig.0000787)
Supplement: S2 File — (ZIP) [file pdig.0000787.s002.zip › S2 Data Raw data files containing unprocessed question inputs and LLM outputs including transcripts of deliberations/STEP3_Question-23-1_Type-MC-NJ.docx]

**Facilitator** **[Round 1]:** A study is being conducted to assess mesothelioma in shipyard workers. A large shipyard firm has provided the asbestos exposure records of all employees during the past 50 years. The health insurer for the workers has provided claims data that documents all chest x-rays and diagnoses of mesothelioma among current workers and retirees. The study enrolled shipyard workers who were diagnosed with mesothelioma and shipyard workers who were not diagnosed with mesothelioma. All subjects in the study had to have chest x‐rays. Which of the following is the best rationale for selecting a comparison group that had chest x-rays? (A) Address confounding (B) Demonstrate causality (C) Minimize ascertainment bias (D) Reduce recall bias

**AI_D:** (1) RESTATING THE QUESTION: To succinctly rephrase the question, we're being asked to discern why a comparison group was selected for a study whose participants all underwent chest x-rays. This study investigates the occurrence of mesothelioma, a type of cancer often linked to asbestos exposure, among shipyard workers, both those diagnosed with the disease and those not diagnosed. (2) PLANNING: Let's examine potential assumptions we might make to answer this question: (a) A comparison group within a study allows for addressing any confounding factors that might lead to inaccurate conclusions; (b) It could demonstrate a causal link between asbestos exposure and mesothelioma; (c) This group may be selected to minimize ascertainment bias, as a chest x-ray is a diagnostic tool for mesothelioma and could lead to a higher detection rate; (d) To mitigate recall bias, as the reliance on workers' memory of their exposure could unintentionally skew data. After reviewing these assumptions, a likely hypothesis here is that the comparison group was chosen to handle potential ascertainment bias. (3) CLINICAL REASONING: Let's consider, in depth, the reasoning for this assumption. Ascertainment bias refers to when information for an outcome or exposure is collected differently across groups, leading to differential misclassification. Having all study subjects undergo chest x-rays—those with and without a mesothelioma diagnosis—is aimed at reducing this bias. If only participants with mesothelioma had received chest x-rays, it could erroneously suggest that there was more mesothelioma in that population due to the higher detection rate. (4) CONCLUDING: Consequently, the study aims to ensure that the rate of disease detection is identical across the different groups. Thus, the rationale behind choosing a comparison group that had chest x-rays is to minimize ascertainment bias. (5) RESPONSE: (C) Minimize ascertainment bias.

**AI_B:** RESTATING THE QUESTION: This study is investigating the occurrence of mesothelioma, a type of cancer often caused by asbestos exposure, in shipyard workers. Asbestos exposure records and health claims data, including chest x-rays and mesothelioma diagnoses, have been provided for all employees over the last 50 years. The participants in the study are shipyard workers, both diagnosed and not diagnosed with mesothelioma, and they all had chest x-rays. The query pertains to the reason for choosing a comparison group that has also undergone chest x-rays. PLANNING: The assumptions underlying this question include: (A) that there might be confounding factors influencing results; (B) one can prove causality between asbestos exposure and mesothelioma; (C) ascertainment bias, which can skew detection of cases, could be minimized; or (D) recall bias could influence the subjects' report of their health history. By having all subjects receive chest x-rays, we can invalidate the recall bias assumption, because the x-ray outcomes are unbiased objective findings and not relying on the worker's memory. Addressing confounding factors and demonstrating causality could be helped to an extent but wouldn't specifically be the main purpose of selecting the group in question. On the other hand, ascertainment bias could be the target of this approach, the bias that affects how data are collected or classified. CLINICAL REASONING: Conducting chest x-rays in both the research and compare group ensures similar conditions under which both sets of data (mesothelioma diagnoses and non-diagnoses) are collected. This would mean that any difference observed between the two groups can be attributed to the presence or absence of mesothelioma and not to differences in data collection. CONCLUDING: Thus, by using chest x-rays for all subjects in the study, irrespective of whether they are diagnosed with mesothelioma or not, the study aims to level the playing field and thus minimize the potential for ascertainment bias. RESPONSE: Therefore, the best rationale for selecting a comparison group that had chest x-rays is to (C) Minimize ascertainment bias.

**AI_A:** 1) RESTATING THE QUESTION: This study revolves around assessing mesothelioma, a type of cancer that emerges from the thin layer of tissue that covers most internal organs, among shipyard workers who were potentially exposed to asbestos over the past 50 years. It requires that all participants, regardless of whether they were diagnosed with mesothelioma or not, have chest x-rays. The question seeks to find out the key reason why a comparison group that underwent chest x‐rays is needed for this study. In this context, addressing confounding means attempting to level out the impact of other factors (confounders) that could influence the outcome. Demonstrating causality means showing the cause and effect relationship, i.e., exposure to asbestos causes mesothelioma. Minimizing ascertainment bias refers to reducing the bias that arises when the disease status isn't equally accurate among the comparison groups. Lastly, reducing recall bias points to mitigating the error because of the different accuracy or completeness of the recollections retrieved by study participants. 2) PLANNING: Let's analyze each assumption. To address confounding, we'd need a comparison group with similar traits except for the potential exposure to asbestos. Demonstrating causality would also require a similar setup, but with a more in-depth examination of the cause-and-effect relationship. Reducing recall bias is about improving the accuracy of participant's memory and this might not have a significant impact as the records of exposure and x-rays are available. Minimizing ascertainment bias seems to be the most relevant as the same mechanism (i.e., chest x-rays) should be used to ascertain disease status in both diagnosed and non-diagnosed participants. 3) CLINICAL REASONING: The use of chest x-rays in both groups equalizes the method of disease detection, therefore minimizing any ascertainment bias. It ensures that the participants' disease statuses are assessed with the same level of accuracy. Even if other biases or confounding variables exist, this consistency in disease assessment should ensure a more reliable comparison. Addressing confounding, demonstrating causality, and reducing recall bias are also important, but these factors don't directly explain the need for chest x-rays in all participants. 4) CONCLUDING: The predominant reason to use chest x-rays in both comparison groups is to minimize ascertainment bias. This method ensures an equal level of accuracy in determining disease status among all participants, thereby establishing a fair baseline for comparison. 5) RESPONSE: Therefore, the best rationale for requiring all participants to have chest x-rays in this mesothelioma study is to (C) Minimize ascertainment bias.

**AI_C:** RESTATING THE QUESTION: The query engages the central issue of why a comparison group that undertook a chest X-ray would be chosen in a study looking at the prevalence of mesothelioma amongst shipyard workers. Mesothelioma is a type of cancer that develops from the thin layer of tissue that covers many of the internal organs (known as the mesothelium). The most common area affected is the lining of the lungs and chest wall. It is well known that asbestos exposure is a major risk factor for mesothelioma. The options provided allude to different possible reasons: to address confounding variables, to demonstrate causality, to minimize ascertainment bias, or to reduce recall bias. PLANNING: In this case, the assumptions are that the cohort of workers who had X-rays would somehow improve the study outcome. We could question whether the X-rays could indeed lead to improved causality detection or minimize biases. A deeper assumption is the connection between X-rays and possible disease detection in this group. Given the scenario, the most pertinent assumption to pursue would be the utility of chest X-rays in reducing ascertainment bias. CLINICAL REASONING: The investigation aims at ascertaining the prevalence of mesothelioma among shipyard workers, notably those exposed to asbestos. All the subjects already had chest X-rays, a crucial diagnostic tool for this disease. Hence, choosing a comparison group that also had chest X-rays would be aimed at ensuring that the method used for disease detection is consistent across both the mesothelioma-diagnosed and non-diagnosed workers. This would help in averting ascertainment bias, an observational bias arising from differential diagnosis procedures or different levels of scrutiny used among different groups. CONCLUDING: In observational studies, it is pivotal to ensure that the process of disease determination is consistent across the study population. If not, we risk introducing an ascertainment bias, leading to unfaithful results. Hence, choosing a comparison group that underwent chest X-rays just like the subjects ensures a level playing ground in disease determination. RESPONSE: The best rationale for choosing a comparison group that had chest X-rays is to minimize ascertainment bias (C).

**AI_E:** 1) RESTATING THE QUESTION: The research at hand is concerned with the investigation of mesothelioma amongst shipyard workers. A study is focusing on shipyard workers both diagnosed with mesothelioma and those not diagnosed with mesothelioma. The commonality between these two groups is that in both cases the workers have had chest x-rays. The question asks, what could be the strongest reason for the researchers to choose a comparison group where all the members have undergone chest x-rays? Mesothelioma is a type of cancer that develops from the thin layer of tissue that covers many of the internal organs (known as the mesothelium), most commonly the lining of the lungs and chest wall. Confounding refers to a distortion of the association between an exposure and an outcome that occurs when the study groups differ with respect to other factors that influence the outcome. Causality refers to the capacity of one variable to influence another. Ascertainment bias refers to the systematic distortion in measuring the true prevalence of a health phenomenon due to the way the data are collected. Recall bias is a systematic error that occurs when participants do not remember previous events or experiences accurately or omit details. 2) PLANNING: The assumption we are making in this question is that having chest x-rays plays a role in the study of mesothelioma in shipyard workers. An alternative assumption could be that the chest x-rays are significant not only in diagnosis but also in minimizing biases in the study. 3) CLINICAL REASONING: Looking at the alternatives provided, we can dissect each one. Addressing confounding (A) could be an option, but the question does not provide any confounding variables that could distort the association being studied. Demonstrating causality (B) could also be an alternative but it does not reason why the chest x-rays are necessary for all participants. Reducing recall bias (D) is a possibility, but less likely because the data was provided by the shipyard firms and health insurer, not the workers themselves thus minimising the probability of inaccuracies in recall. The most compelling option here is minimizing ascertainment bias (C). This bias might arise if the diagnosis of mesothelioma was ascertained differently between the shipyard workers diagnosed with and without mesothelioma. To prevent this, having a common method of diagnosis or confirmation (chest x-rays) for all shipyard workers can help to ensure that the detection in both groups was consistent. 4) CONCLUDING: Thus, in a study design, it is vital to maintain consistencies where possible to ensure reliable and valid results. In this case, having all subjects undergoing chest x-rays would allow researchers to be confident that mesothelioma's presence or absence was not due to the inappropriate allocation or availability of diagnostic resources. 5) RESPONSE: Therefore, selecting a comparison group that had chest x-rays is best rationalized by the need to minimize ascertainment bias (C).

**Facilitator** The question pertains to a study investigating mesothelioma, a type of cancer often linked to asbestos exposure, among shipyard workers. The study includes both workers diagnosed with mesothelioma and those not diagnosed, all of whom have undergone chest x-rays. The query seeks to understand the primary reason for selecting a comparison group that also underwent chest x-rays. The assumptions underlying this question include the potential for confounding factors, the ability to demonstrate causality between asbestos exposure and mesothelioma, the possibility of minimizing ascertainment bias, and the potential influence of recall bias. Addressing confounding factors and demonstrating causality are important aspects of any study, but they do not directly explain the need for chest x-rays in all participants. Recall bias is less likely to be a significant issue in this study, as the data was provided by the shipyard firms and health insurer, not the workers themselves, thus minimizing the probability of inaccuracies in recall. The most compelling rationale for having all participants undergo chest x-rays is to minimize ascertainment bias. Ascertainment bias refers to a systematic distortion in measuring the true prevalence of a health phenomenon due to the way the data are collected. By ensuring that all participants, regardless of their disease status, undergo the same diagnostic procedure (chest x-rays), the study can ensure a consistent method of disease detection across all groups. This approach reduces the risk of differential misclassification and ensures that any observed differences between the groups can be attributed to the presence or absence of mesothelioma, rather than differences in data collection or diagnosis procedures. Therefore, the consensus among the responses is that the best rationale for selecting a comparison group that had chest x-rays is to minimize ascertainment bias (C).
